# EDITOR’S MESSAGE / MESSAGE DE LA RÉDACTION

**DOI:** 10.29173/jchla29944

**Published:** 2026-04-01

**Authors:** Ashley Farrell

**Affiliations:** JCHLA/JABSC Editor

Welcome to our spring issue of the JCHLA/JABSC. I am very happy to say goodbye to winter, and I’m sure many of you are too. Our issue opens with an announcement from the CHLA/ABSC 2026 Conference Chair regarding this year’s upcoming conference in beautiful Québec City. This year, the CHLA/ABSC will mark its 50th anniversary, celebrated under the theme, “Honouring the Past, Shaping the Future: 50 Years of Health Expertise.”

In this issue, we have two book reviews. Adoranti considers *The new instruction librarian: a workbook for trainers and learners*, and Rutherford examines *Legislative advocacy and public policy work for academic and research library workers: perspectives and strategies*.

Our issue concludes with a product review from Mason and An on GlobalData Intelligence Center (Medical), which considers the resource from the intersection of health and business librarianship.

We hope you enjoy this issue of JCHLA/JABSC. If you are submitting your work to the CHLA/ABSC 2026 Conference, we also invite you to consider submitting your work to the JCHLA/JABSC. For submission help, please see our Author Guidelines. We’d love to see your research highlighted in our journal.

**Ashley Farrell**

*JCHLA/JABSC Editor*

*Email:* editor@chla-absc.ca

Bienvenue au numéro de printemps du JABSC/JCHLA. Je suis très heureuse de dire au revoir à l’hiver, et je suis certaine que beaucoup d’entre vous le sont aussi. Notre numéro débute par une annonce de la présidente du congrès ABSC/CHLA 2026 concernant le congrès qui se tiendra cette année dans la magnifique ville de Québec. Cette année, l’ABSC/CHLA souligne son 50e anniversaire, célébré sous le thème : « Honorer le passé, façonner l’avenir : 50 ans d’expertise en santé ».

Dans ce numéro, nous présentons deux critiques de livres. Adoranti examine *The new instruction librarian: a workbook for trainers and learners*, et Rutherford analyse *Legislative advocacy and public policy work for academic and research library workers: perspectives and strategies*.

Nous terminons ce numéro par une critique de produit signée Mason et An portant sur GlobalData Intelligence Center (Medical), qui examinent cette ressource à l’intersection de la bibliothéconomie de la santé et des affaires.

J’espère que vous apprécierez ce numéro du JABSC/JCHLA. Si vous soumettez vos travaux au congrès de l’ABSC/CHLA 2026, nous vous invitons également à envisager de soumettre un article au JABSC/JCHLA. Pour obtenir de l'aide concernant la soumission, veuillez consulter les Directives aux auteurs. Nous serions ravis de mettre votre recherche en valeur dans notre revue.

**Ashley Farrell**

*Directrice de la rédaction JABSC/JCHLA*

*Courriel:* editor@chla-absc.ca

